# Ptychographic lens-less birefringence microscopy using a mask-modulated polarization image sensor

**DOI:** 10.1038/s41598-023-46496-z

**Published:** 2023-11-07

**Authors:** Jeongsoo Kim, Seungri Song, Hongseong Kim, Bora Kim, Mirae Park, Seung Jae Oh, Daesuk Kim, Barry Cense, Yong-min Huh, Joo Yong Lee, Chulmin Joo

**Affiliations:** 1 Department of Mechanical Engineering, Yonsei University, Seoul, 03722 Republic of Korea; 2 Department of Ophthalmology, Asan Medical Center, University of Ulsan College of Medicine, Seoul, 05505 Republic of Korea; 3 Department of Radiology, College of Medicine, Yonsei University, Seoul, 03722 Republic of Korea; 4 YUHS-KRIBB Medical Convergence Research Institute, Seoul, 03722 Republic of Korea; 5 Department of Mechanical System Engineering, Jeonbuk National University, Jeonju, 54896 Republic of Korea; 6 Department of Electrical, Electronic and Computer Engineering, The University of Western Australia, Perth, WA 6009 Australia; 7 Department of Biochemistry and Molecular Biology, College of Medicine, Yonsei University, Seoul, 03722 Republic of Korea

**Keywords:** Polarization microscopy, Lasers, LEDs and light sources

## Abstract

Birefringence, an inherent characteristic of optically anisotropic materials, is widely utilized in various imaging applications ranging from material characterizations to clinical diagnosis. Polarized light microscopy enables high-resolution, high-contrast imaging of optically anisotropic specimens, but it is associated with mechanical rotations of polarizer/analyzer and relatively complex optical designs. Here, we present a form of lens-less polarization-sensitive microscopy capable of complex and birefringence imaging of transparent objects without an optical lens and any moving parts. Our method exploits an optical mask-modulated polarization image sensor and single-input-state LED illumination design to obtain complex and birefringence images of the object via ptychographic phase retrieval. Using a camera with a pixel size of 3.45 μm, the method achieves birefringence imaging with a half-pitch resolution of 2.46 μm over a 59.74 mm^2^ field-of-view, which corresponds to a space-bandwidth product of 9.9 megapixels. We demonstrate the high-resolution, large-area, phase and birefringence imaging capability of our method by presenting the phase and birefringence images of various anisotropic objects, including a monosodium urate crystal, and excised mouse eye and heart tissues.

## Introduction

Since the invention of the first compound microscope, several hardware improvements, including optical components and image sensors, have been made over the years. However, its basic design consisting of multiple lenses to form an optical image of an object has not changed significantly. This conventional imaging system is beset by the inherent trade-offs between the spatial resolution and field-of-view (FoV), limiting their space–bandwidth product (SBP), which can be calculated as the FoV divided by the square of the spatial resolution^1^. In other words, one can only image the fine details of an object with high resolution in a small region. Because imaging systems require a large SBP to handle multiple applications, the conventional imaging design relies on complicated optical and mechanical architectures to increase the SBP, resulting in a bulky and expensive imaging setup.

In recent years, as advanced computational algorithms have been developed and computational resources became more powerful, various computational microscopy techniques were developed to overcome the SBP limitation of conventional imaging systems^2–5^. Lens-less microscopy is one of the representative computational imaging platforms that were developed to address the SBP limitation, while enabling a “lean” optical architecture^6–9^. Because the final image can be obtained with unit magnification by placing the object directly on the image sensor, the imaging FoV is only limited by the size of the image sensor, typically a few millimeters, and the resolution is determined by the pixel size of the imaging sensor^9^. Moreover, various pixel-super resolution methods can be employed to further enhance the resolution, using measurement diversities based on multiple object heights^10,11^, object/sensor translation^12,13^, multiple wavelengths^14,15^, etc. Synthetic aperture-based lens-less imaging technology also enables high-resolution imaging by enlarging the effective numerical aperture (NA) using angle varied illumination^16^. The lens-less microscope can also retrieve the complex information of the object from intensity-only measurements via back projection and a phase retrieval algorithm. Taking advantage of these features, namely high information throughput, small form-factor, and cost-effectiveness, various imaging modalities such as phase and fluorescence imaging have been demonstrated in a lens-less platform^17,18^, and numerous applications have been proposed, including cell observation^19^, disease diagnosis^6^, and air quality monitoring^20^.

Birefringence refers to the polarization-dependent refractive index of optically anisotropic materials, resulting from the ordered arrangement of microstructures within the materials^21,22^. Birefringence can be found in a myriad of natural and engineered materials, including collagen, reticulin^23^, and various kinds of synthetic two-dimensional materials^24^. This intrinsic property has thus been broadly studied for various applications such as material inspection^25–27^ and biomedical diagnosis^28–30^. Polarization light microscopy (PLM) enables to assess anisotropy properties such as phase retardation and optic-axis orientation in transparent materials. The operation of PLM entails capturing multiple images with mechanical rotations of the polarizer and analyzer in a conventional optical microscope, which is a relatively complex process. Furthermore, PLM, like other conventional microscopes, suffers from limited SBP. To address these limitations, various imaging methods like digital holographic microscopy^31^, ptychography^32^, single-pixel imaging^33^, differential phase-contrast microscopy^34^, and lens-less holographic microscopy^35,36^ have been explored for birefringence imaging. Among them, lens-free holographic polarization microscopy enables large-area birefringence imaging in a lens-free manner^35^, but two sets of raw holograms must be taken with illuminations in two different polarization states, which requires precise image alignment of the recorded images. Recently, a promising approach has been proposed that combines deep learning with lens-free holographic polarization microscopy to achieve single-shot birefringence imaging, although it requires training with a large number of datasets^37^.

Here, we present a form of lens-less polarization-sensitive (PS) microscopy, which allows for inertia-free, large-area, complex and birefringence imaging. Our polarization-sensitive ptychographic lens-less microscope (PS-PtychoLM) adopts a high-resolution, large-FoV imaging capability of a mask-modulated ptychographic imager^38,39^, while quantitatively measuring the birefringence properties of transparent samples with a single-input-state illumination and polarization-diverse imaging system. Compared to conventional polarization imaging techniques, our PS-PtychoLM does not involve any mechanical rotation of the polarizer/analyzer and translation of object or light source, which makes the system more robust and easier to operate. Bai et al.^40^ has previously demonstrated a computational PLM capable of inertia-free birefringence imaging for transparent anisotropic crystals using a polarization image sensor. However, the method is built on an optical microscope with bulky optics, and does not provide complex information (i.e., amplitude and phase). In contrast, our method is lens-less, and provides both complex and birefringence information of objects over a large FoV. PS Fourier ptychographic microscopes have been introduced^41, 42^, but still demonstrated in the platforms of optical microscopes, which is distinct from our lens-free implementation.

We employ a Jones-matrix analysis to formulate the PS-PtychoLM image reconstruction process and evaluate various pixel demosaicing methods to suggest the optimum demosaicing scheme in the mask-modulated ptychographic platform. We demonstrate high-accuracy PS imaging capability over a large-FoV by presenting the birefringence images of various anisotropic objects, including a monosodium urate (MSU) crystal, mouse eye and heart tissue sections.

## Results

### PS-PtychoLM optical setup

**Figure 1:**
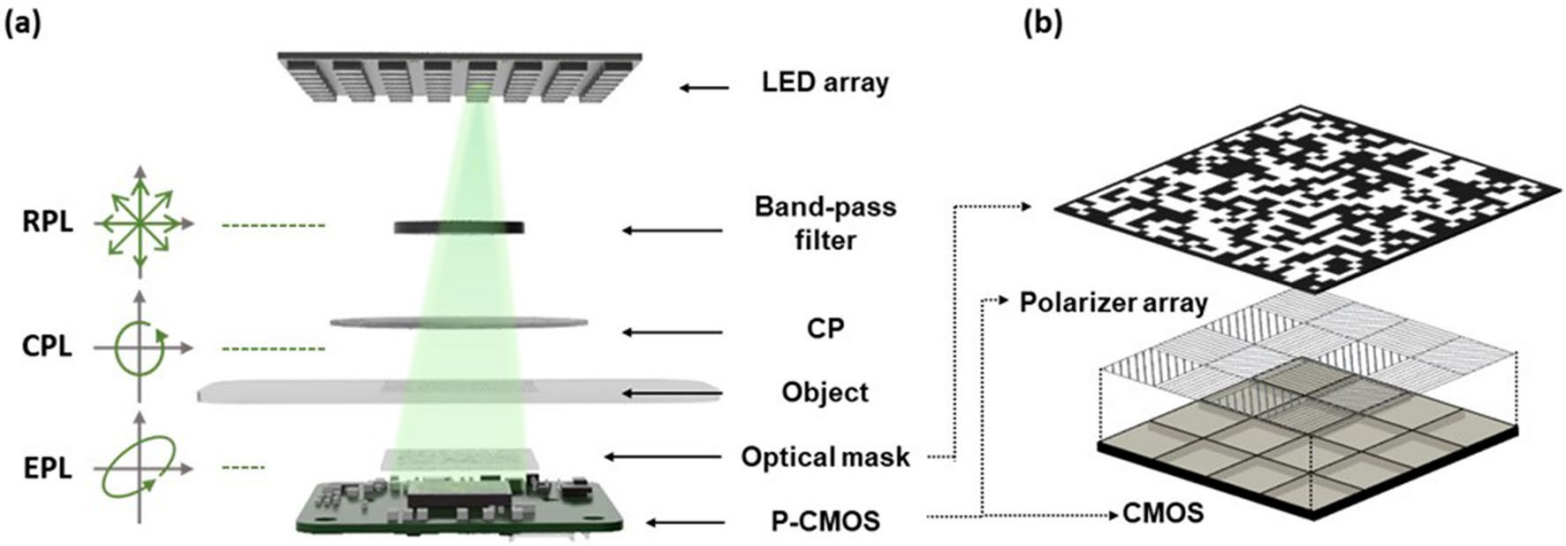
PS-PtychoLM. (**a**) Schematic of polarization-sensitive ptychographic lens-less microscope (PS-PtychoLM). A programmable light-emitting diode (LED) array is turned on sequentially to generate angle-varied illumination. Unpolarized LED light passes through a band-pass filter and circular polarizer (CP) to be circularly polarized and to illuminate an object. (**b**) The light transmitted through the object is intensity modulated by an optical mask, and then recorded through four different polarization channels of a polarization camera (P-CMOS). (RPL: randomly polarized light, CPL: circularly polarized light, EPL: elliptically polarized light).

Our PS-PtychoLM was built on a mask-modulated ptychographic imager^38,39^, which provides inertia-free complex image reconstruction with a programmable LED array. A schematic of the PS-PtychoLM is depicted in Fig. 1. We employed a custom-built programmable LED array for angle-varied illumination. The LED array was composed of high-power LEDs (Shenzhen LED color, APA102-202 Super-LED, center wavelength = 527 nm, full-width at half-maximum bandwidth ~ 30 nm) and featured a 4 mm pitch with 9 × 9 elements that can be controlled independently. The central 9 × 9 elements of the LED array were used, and a total of 81 raw intensity images were captured for each LED illumination. The distance from the LED array to the object was set to be ~ 400 mm, providing illumination angles of -2.29 to 2.29°. Prior to illuminating an object, the LED light passed through a band-pass filter (Thorlabs, FL514.5-10, center wavelength = 514.5 nm, full-width at half-maximum bandwidth = 10 nm) to increase temporal coherence, after which the light was circularly polarized using a zero-order circular polarizer (Edmund optics, CP42HE). If the object is optically anisotropic, the light passing through the object becomes elliptically polarized. The transmitted light was then intensity modulated by a binary amplitude mask (Fig. 1b). The mask was fabricated by coating a 1.5 mm thick soda-lime glass slab with chromium metal with a random binary pattern that was employed in Ref. 38,39. The transparent-to-light-blocking area ratio of the optical mask was designed to be one and the pattern had a feature size of 27.6 μm. The modulated light was then captured using a board-level polarization-sensitive complementary metal–oxide–semiconductor image sensor (P-CMOS, Lucid Vision Labs, PHX050S1-PNL) with a pixel resolution of 2048 × 2448 and a pixel size of 3.45 μm, allowing for a FoV of 7.07 mm × 8.45 mm. The camera was equipped with four different directional polarization filters (0°, 45°, 90°, and 135°) on the image sensor for every four pixels, allowing the information of each polarization state to be captured simultaneously (Fig. 1b). PS-PtychoLM could thus acquire polarization images along a specific orientation required for computing the birefringence distribution without mechanical rotation of the polarizers or variable retarder.

### Numerical simulation

**Figure 2:**
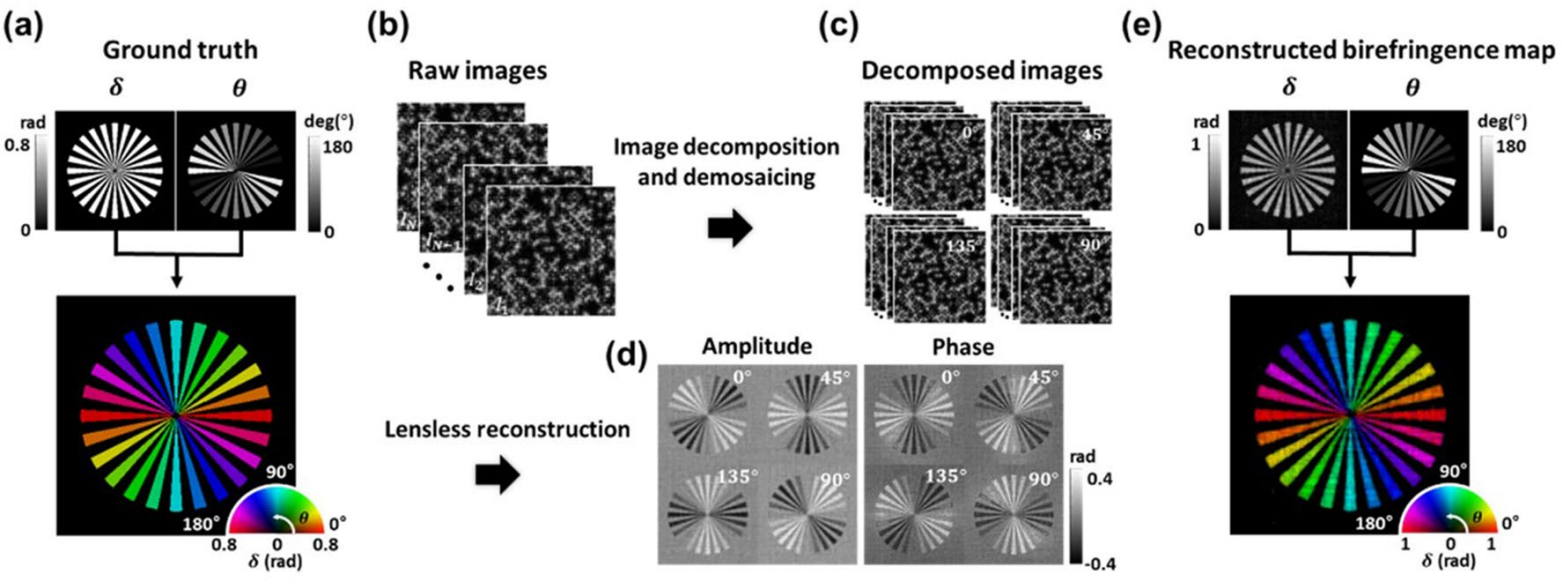
PS-PtychoLM birefringence map reconstruction procedure and simulation results. (**a**) Ground truth birefringence information of a simulation target. (**b**, **c**) PS-PtychoLM captures raw images under angle-varied LED illuminations, and each recorded image is decomposed into the images in four polarization channels. The missing pixel information in each image is interpolated using a polarization demosaicing scheme. (**d**) The amplitude and phase information of each polarization channel are then reconstructed using a ptychographic reconstruction algorithm, and further used to obtain phase retardation and optic-axis orientation maps of the object based on Jones-matrix analysis. (**e**) Obtained birefringence information can be jointly presented using a pseudo-color map that encodes retardation and optic-axis orientation with intensity and color, respectively.

Figure 2 outlines the PS-PtychoLM image reconstruction procedure and numerical simulation results of a simulated birefringence object. We considered a Siemens birefringent object, of which each sector form was characterized by a phase retardation of π/4 and their optic axes oriented along the longer sides. The corresponding birefringence map is presented by a pseudo-color map to denote optic-axis orientation (θ) and retardation (δ) with color and intensity, respectively (Fig. 2a). The PS-PtychoLM measurements were numerically performed (Fig. 2b). The recorded images by the P-CMOS under angle-varied LED illuminations were first decomposed into four images at different polarization channels. The resultant images were then processed for polarization demosaicing to estimate missing polarization information at the neighboring pixels (Fig. 2c). For the demosaicing, we used Newton's polynomial interpolation model^43^ because it was found to recover images with high fidelity in both low- and high-frequency features, compared to other schemes (see Methods). The amplitude and phase images of each polarization channel were then reconstructed using a ptychographic reconstruction algorithm (Fig. 2d)^44^ (Sects. 1 and 2 in the Supplementary Information) and further used to obtain phase retardation and optic-axis orientation of the birefringent object based on the Jones-matrix analysis. The reconstructed birefringence information is presented in Fig. 2e, which agrees with the ground truth map (Fig. 2a). Details of the forward imaging model and reconstruction algorithm are provided in the Supplementary Information Sects. 1 and 2.

### Experimental demonstration: MSU crystals

**Figure 3:**
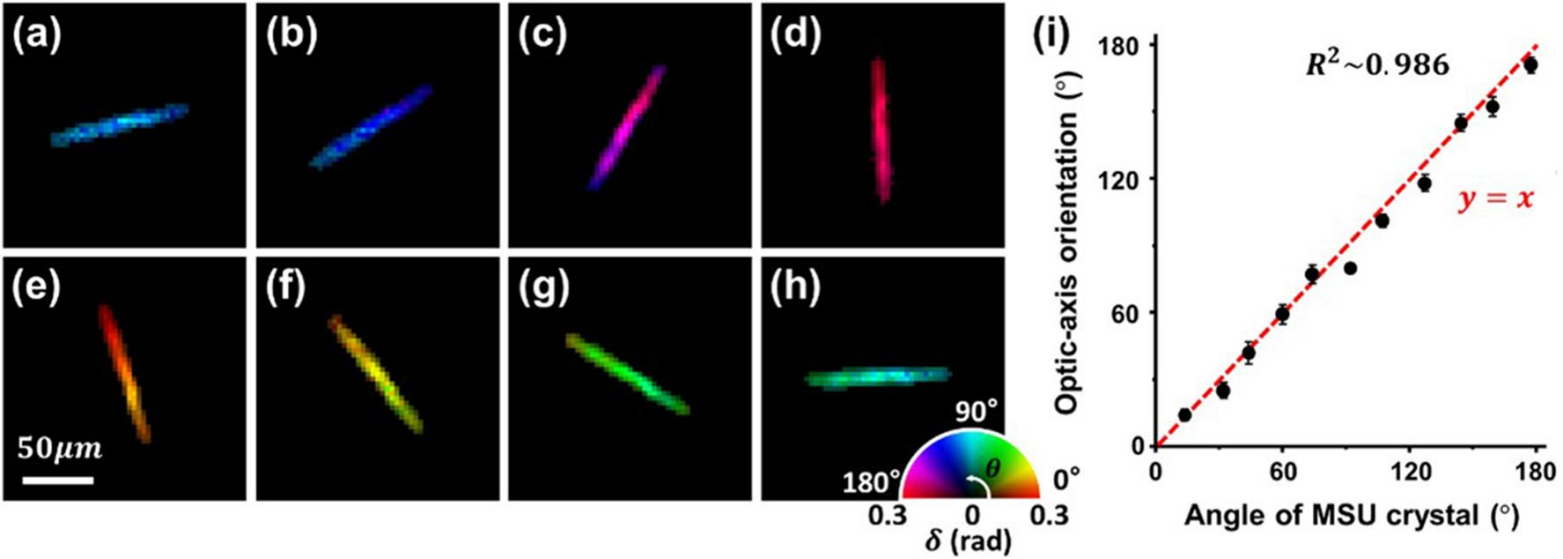
Validation of PS-PtychoLM optic-axis orientation measurements. (**a**–**h**) Birefringence maps of a single MSU crystal at different rotation angles (15°,33°,61°,93°,108°,128°,145°, and 178°, respectively). (**i**) Optic-axis orientation of the MSU crystal measured through rotation at approximately 15° intervals.

We then experimentally performed imaging of monosodium urate (MSU) crystals (InvivoGen Co., tlrl-MSU) immersed in phosphate-buffered saline to validate the birefringence imaging capability of the PS-PtychoLM. The MSU crystals are needle-shaped birefringent crystals with strong negative birefringence, i.e., the fast axis is oriented along the axial direction of the crystals^35^. Following the PS-PtychoLM reconstruction, we selected and analyzed a single MSU crystal to verify optic-axis orientation measurements. Figure 3a–h present representative birefringence maps of the single MSU crystal at various rotation angles. Figure 3i shows a comparison of the optic-axis orientation measured with PS-PtychoLM and that obtained manually with the NIH ImageJ program. The coefficient of determination (R^2^) was measured to be ~ 0.986, and the phase retardation was found to be ~ 0.272 rad, regardless of the rotation angle. The standard deviation of optic-axis orientation distribution was calculated over the pixels in the MSU crystal (104 pixels) for each angle and is indicated by the error bars in Fig. 3i. To evaluate the PS-PtychoLM accuracy in the determination of phase retardation, we used the information in the literatures to estimate the phase retardation of the MSU crystals. Using the refractive index information in Park et al.^45^ $(n\approx 1.41)$ and measured phase maps in our reconstruction result, we first estimated the diameter of the MSU crystal to be 0.236 μm. The phase retardation was then determined to be 0.288 rad based on the birefringence information of the crystals in Zhang et al.^35^ $(\Delta n=0.1)$. Compared with the PS-PtychoLM measurement (i.e., ~ 0.272 rad), the relative error was found to be 5.66%.

### Experimental demonstration: biological tissue samples

**Figure 4:**
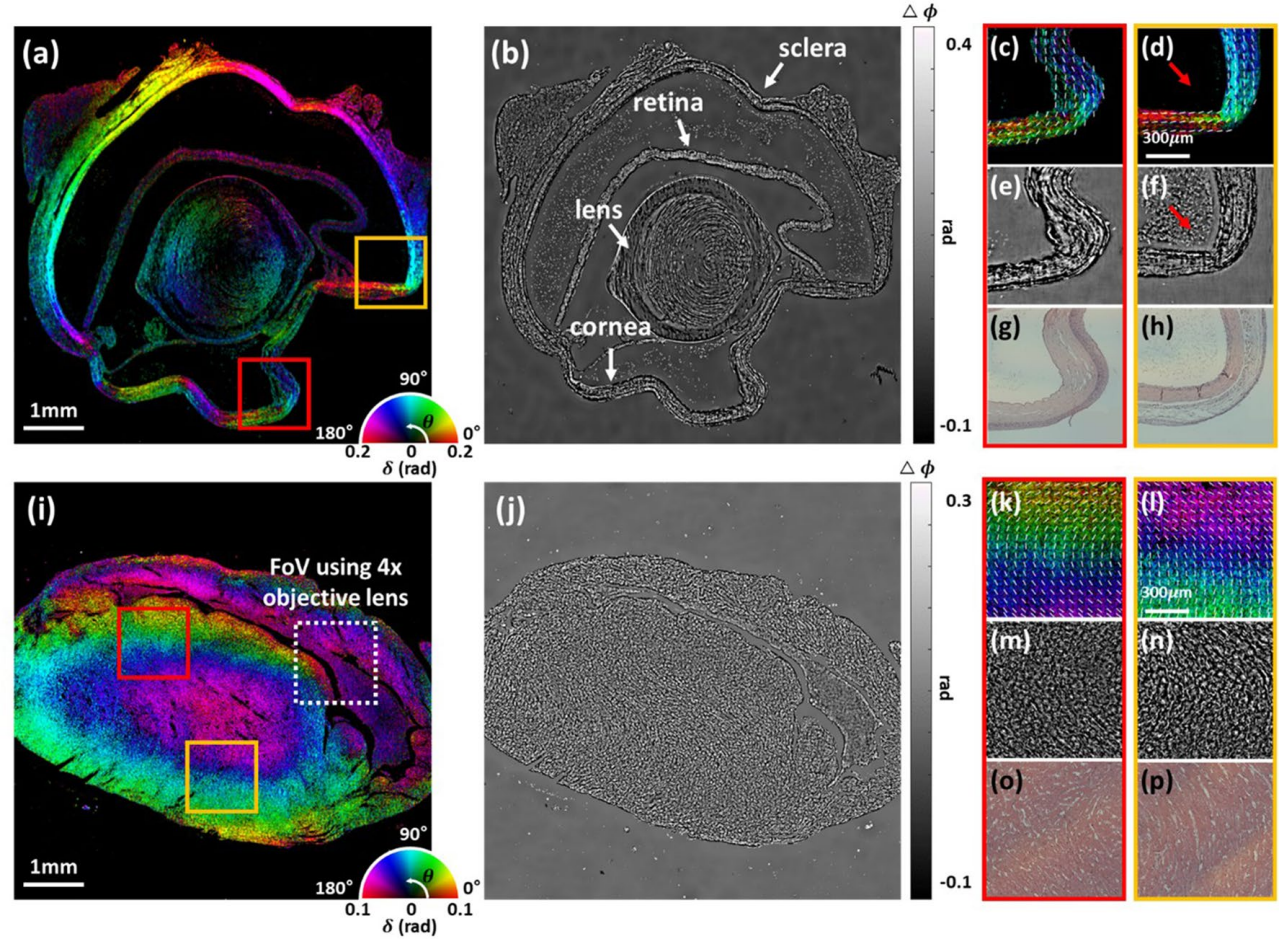
PS-PtychoLM imaging results of mouse eye (**a**–**h**) and heart tissue specimens (**i**–**p**). (**a**, **i**) Quantitative birefringence maps of the mouse eye and heart tissues over the entire FoV. (**b**, **j**) Quantitative phase images of the mouse eye and heart tissues over the entire FoV. (**c**–**f**) Enlarged birefringence and phase images of the parts marked with red and orange boxes in (a), respectively. (**g**, **h**) Hematoxylin and eosin (H&E) staining images of the parts (**c**, **d**) captured with an optical microscope (Nikon, ECLIPSE Ti, 0.2NA). (**k**–**n**) Enlarged birefringence and phase images of the parts marked with red and orange boxes in (**i**), respectively. (**o**, **p**) H&E staining images of the parts (**k**, **l**) captured with an optical microscope (Nikon, ECLIPSE Ti, 0.2NA). Overlaid white short lines on (**c**, **d**, **k**, and **l**) indicate the mean optic-axis orientation evaluated over a small region (70 μm × 70 μm).

In order to demonstrate large-FoV PS-PtychoLM imaging capability for biological specimens, we further performed PS-PtychoLM imaging of mouse eye and heart tissues. Paraffin-embedded mouse eye and heart tissue sections were obtained from Asan Medical Center and Yonsei Severance Hospital, Seoul, Republic of Korea, respectively. All experimental procedures were carried out in accordance with the guidelines of the Institutional Animal Care and Use Committees which were approved by Asan Institute for Life Science and Yonsei University College of Medicine. We de-paraffinized the tissue sections (the eye and heart were sectioned at 10 μm and 5 μm thick, respectively) using hot xylene, placed them onto a microscope glass slide, and performed PS-PtychoLM imaging. Shown in Fig. 4a,b are the large-FoV quantitative birefringence and phase images of the mouse eye section, respectively. The phase image was obtained by evaluating the mean of the phases obtained at the four polarization channels. Some parts of the eye, including the cornea and sclera, are formed of a stack of orientated collagen fibers, which result in birefringence with its optic-axis orientated along the fibers^46,47^. Figure 4c-f show the magnified birefringence and phase images of the parts marked with red and orange boxes in Fig. 4a, which correspond to the cornea and sclera, respectively. Visualization of the optic-axis orientation was enhanced by overlaying short white lines that indicate the mean optic-axis orientation evaluated over a small region (70 μm × 70 μm). Through the ptychographic phase retrieval algorithm, PS-PtychoLM provides complex and birefringence information of the specimen, which can be used to measure various features of the objects. The phase information provides optical path-length distribution of the sample, while the birefringence map allows for interrogating phase retardation and optic-axis orientation. This feature is manifested in Fig. 4d and f in that the cellular structures observed in the phase image are not visualized in the corresponding birefringence image, as marked with red arrow. Figure 4g,h are the images captured with a conventional microscope (Nikon, ECLIPSE Ti, 0.2 NA) after the samples were stained with hematoxylin and eosin (H&E). It is clearly observed that the fiber arrangement observed in Fig. 4g,h greatly matched the optic-axis orientation obtained with PS-PtychoLM.

Figure 4i,j present the birefringence and phase images of the mouse heart section, along with the enlarged images (Fig. 4k,l for birefringence and Fig. 4m,n for phase) of the parts marked with red and orange boxes in Fig. 4i. Because fibrous tissues found in the myocardium exhibit optical birefringence, the optic-axis information may be used to infer myocardium orientation^48^. Figure 4o,p are the corresponding H&E images obtained from the optical microscope (Nikon, ECLIPSE Ti, 0.2 NA); these images correspond to the same area depicted in Fig. 4k and l, respectively. The alignment direction of the myocardium observed in the H&E image is consistent with the reconstructed optic-axis orientation in the PS-PtychoLM images. Myofiber disorganization compromises normal heart function and is associated with various cardiovascular diseases such as myocardial infarction. Note that, the boxed area marked with a white dotted line in Fig. 4i indicate the FoV obtainable with a 4 × objective lens, which provides the same spatial resolution as the PS-PtychoLM. One can see that in this limited FoV, it is difficult to obtain a comprehensive information of the myocardium alignment direction^49^ over the large tissue specimens. These results suggest that, with further improvement, PS-PtychoLM may serve as a viable PS imaging tool for large-scale biological specimens and disease diagnosis.

## Conclusion

In summary, we described a computational lens-less birefringence microscope by combining the inertia-free, high-resolution, large-area imaging capability of the mask-modulated ptychography with single-input-state polarization-sensitive imaging setup. The proposed PS-PtychoLM could produce complex-valued information on each polarization channel of the image sensor, and the information was further used to obtain a 2D birefringence map over the entire sensor surface. Our method featured a half-pitch resolution of 2.46 μm across an FoV of 7.07 mm × 8.45 mm, which corresponds to 9.9 megapixels in SBP.

Several features should be noted in the reported platform. We employed the mask-modulated ptychographic configuration with angle-varied LED illumination to achieve inertia-free ptychographic birefringence imaging. The programmable LED array enabled simple, cost-effective, and motion-free operation of PS-PtychoLM, but its short coherence both in spatial and temporal domains and low light-throughput compromised the spatial resolution and imaging speed. We used a bandpass filter in the illumination path to improve the temporal coherence, but the spatial coherence could not be improved because either the use of smaller LEDs or larger distance between the LEDs and object compromised the light throughput. The imaging throughput in our prototype is limited largely by low light-throughput of the LEDs. Instead of LEDs, coherence light sources such as laser or laser diodes can certainly be employed. These light sources provide directed beams with higher spatial and temporal coherence, but in such cases, either the illumination light or object must be scanned or translated to obtain measurement diversity, which is required for ptychographic phase retrieval. Note that various implementations of ptychographic microscopy are summarized in Wang et al.^50^. In our study, we aimed to demonstrate a cost-effective and robust imaging platform and thus employed the LED array as the illumination source instead of the aforementioned solutions. It should be noted that mask structures that can divert high spatial frequency information scattered from the object into the image sensor would also help improving the spatial resolution. Recent studies reported on various mask configurations for high-resolution lens-less imaging, and among them, mask structures with broad spatial frequency responses were found to produce superior image reconstruction results^17,51,52^. Optimized mask designs in the platform of PS-PtychoLM are underway.

Measurement uncertainty of birefringence properties is highly dependent on the precision of intensity information reconstructed in each polarization channel. The theoretical measurement uncertainty of birefringence properties can be quantified by evaluating the variances of optic-axis orientation $(\langle {\sigma }_{\theta }^{2} \rangle )$ and phase retardation $(\langle {\sigma }_{\delta }^{2}\rangle )$ fluctuations, which were found to be $\langle {\sigma }_{\theta }^{2}\rangle \sim \langle {\Delta Q}^{2}\rangle /8{Q}_{0}^{2},$ and $\langle {\sigma }_{\delta }^{2}\rangle \sim \langle {\Delta Q}^{2}\rangle /2\left(1-{Q}_{0}^{2}\right),$ with ${Q}_{0}^{2}={Q}_{1}^{2}+{Q}_{2}^{2}$ and ${\Delta Q}^{2}=\Delta {Q}_{1}^{2}+\Delta {Q}_{2}^{2}$. Here, $\Delta {Q}_{1}$ and $\Delta {Q}_{2}$ are the fluctuations of ${Q}_{1}$ and ${Q}_{2}$ respectively (see Eq. (8,9) in Methods). The uncertainty in the intensity information is influenced by various factors, including image sensor characteristics such as dark/readout noise and angular response to incident light, unwanted spatial phase noise incurred in the pixel interpolation step, the intensity fluctuations of illumination LEDs, and misalignment in the LED illumination angles. Hence, the use of image sensors with larger well capacities and low noise would help to improve the measurement precision. Moreover, image sensors with smaller pixel sizes enable finer spatial sampling, which mitigates any errors during the interpolation stage. For high-resolution image reconstruction, one should also consider the angular response of the image sensor to different angles of incident light. Recently, Jiang et al.^17^ developed an imaging model that considers the angular response of the image sensor. A similar model can be implemented in the PS-PtychoLM imager to improve the imaging performance. In terms of the light source, the use of power-stable light sources is highly desired to alleviate the issues with intensity noises from the light source. Li et al.^53^ recently introduced a computational method that performs angle calibration and corrects for LED intensity fluctuations in the platform of lens-less ptychography. Employment of such strategies to correct for potential noise sources is likely to improve the detection performance of the PS-PtychoLM imager.

In our PS-PtychoLM implementation, we employed binary mask-assisted ptychographic phase retrieval to obtain complex information of the object. Instead of the binary mask, several recent studies reported on using other random structures such as diffusers with micro-/nano- features to achieve superior resolution^8,17^. However, we note that with the random diffuser in our setup, the object information could not be accurately obtained. Our prototype employed a pixelated polarization image sensor, and it is thus required to decompose the captured image into the ones in different polarization channels and perform pixel interpolation through demosaicing methods. However, the random diffuser significantly scrambles the propagation wavefront of the object wave, and thus information in the missing pixels could not be correctly interpolated by the demosaicing schemes. We numerically investigated the mean squared errors (MSEs) between the information acquired with a full pixel-resolution camera (i.e., without pixel demosaicing method) and the one obtained with pixelated polarization camera and pixel demosaicing. The measured MSE with the random diffuser was found to be ~ 3 × larger than that with the binary mask. Detailed simulation results of PS-PtychoLM imaging with diffuser and binary mask are provided in the Supplementary Information Sect. 4. We also experimentally validated that the use of a binary mask provided an accurate estimation of polarization information in all the detection channels, thus resulting in accurate birefringence maps. This problem can certainly be alleviated by using a non-polarized full pixel-resolution camera, but it would require mechanical rotation of polarizer/analyzer to reconstruct birefringence maps.

In terms of the mask pattern, we utilized a binary amplitude mask with 50% open channels. Optimal binary mask designs for rapid and robust phase recovery are certainly the subject of future research, as also noted by Ref. 39. The influence of the mask design parameters (e.g., feature size and distribution) on the image recovery performance is being investigated. The results are expected to provide useful guidelines for binary mask designs in the mask-assisted ptychographic imaging systems.

We expect that our method would have a broad range of applications in material inspections and various quantitative biological studies. For example, large-FoV, high-resolution birefringence imaging can be used to inspect internal features (e.g., molecular orientation of the liquid crystal layer between two substrates) of liquid crystal-based devices^54^ and to detect defects of semiconductor wafers in the manufacturing process^25^. In addition, birefringence imaging can be used in several biomedical applications such as for malaria detection^41,55^, brain slide imaging^56^, retina imaging^57,58^, cancerous cell differentiation^59,60^ and bulk tissue characterization^61^. On a technical note, PS-PtychoLM can be extended to depth-multiplexed birefringence imaging by considering the wave propagation through the multilayered structures. Each layer can be regarded as a thin anisotropic material, and birefringence information of each layer can be updated iteratively via ptychographic algorithms. This platform would enable high-throughput optical anisotropy imaging of stacked specimens. Note that similar approach has been demonstrated for depth-multiplexed phase imaging^62^. Our demonstration is limited to imaging only thin and transparent samples. However, implementation of the multi-slice beam propagation (MBP) model^63,64^ on the lens-less imaging platform would enable imaging of thick multiple scattering samples through a combination with illumination engineering methods (e.g., angle scanning). Recently, Song et al.^65^ introduced tomographic birefringence microscopy based on vectorial MBP model and gradient decent method. Relevant forward imaging model and image reconstruction algorithms may be adopted and integrated into our PS-PtychoLM platform, generating lens-less tomographic birefringence imager.

## Methods

### Jones-matrix analysis for PS-PtychoLM

The Jones-matrix analysis was used to formulate the PS-PtychoLM image reconstruction process, in which the polarization state of light is represented by a Jones vector, and the optical elements, including specimens, are represented by Jones matrices^66,67^. Jones matrix for a thin sample $\left({J}_{s}\right)$ can be represented with a phase retardance magnitude (δ) and an optic-axis orientation (θ) as:

1 $$J_{s} = RDPR^{ - 1} ,$$

where $$R=\left[\begin{array}{cc}cos\theta & -sin\theta \end{array}; \begin{array}{cc}sin\theta & cos\theta \end{array}\right]$$ is a rotation matrix defined by optic-axis orientation θ, $$P = \left[ {\begin{array}{*{20}c} {e^{i\delta /2} } & 0 \\ \end{array} ; \begin{array}{*{20}c} 0 & {e^{ - i\delta /2} } \\ \end{array} } \right]$$ is the phase retardation matrix, and $D$ is the diattenuation matrix which is negligible in thin specimens. As a result, the Jones matrix for thin specimens can be formulated as:

2 $$J_{s} = e^{{\frac{i\delta }{2}}} \left[ {\begin{array}{*{20}c} {\cos^{2} \theta + e^{ - i\delta } \sin^{2} \theta } & {\left( {1 - e^{ - i\delta } } \right)\sin \theta \cos \theta } \\ {\left( {1 - e^{ - i\delta } } \right)\sin \theta \cos \theta } & {\sin^{2} \theta + e^{ - i\delta } \cos^{2} \theta } \\ \end{array} } \right].$$

In addition, the Jones matrix for the linear polarizer on the detector $\left( {J_{d,\psi } } \right)$ along ψ direction $\left( {\psi = 0^{^\circ } ,45^{^\circ } ,90^{^\circ } ,and \;135^{^\circ } } \right)$ is expressed as:

3 $$J_{d,\psi } = \left[ {\begin{array}{*{20}c} {\cos^{2} \psi } & {\sin \psi \cos \psi } \\ {\sin \psi \cos \psi } & {\sin^{2} \psi } \\ \end{array} } \right].$$

Light field output ($E_{out,\psi }$) measured at each polarization channel through our imaging system can be represented as:

4 $$E_{out, \psi } = J_{d,\psi } J_{s} E_{in} = \sqrt{\frac{I}{2}} \left[ {\begin{array}{*{20}c} {J_{\psi , xx} } & {J_{\psi , xy} } \\ {J_{\psi , xy} } & {J_{\psi , yy} } \\ \end{array} } \right]\left[ {\begin{array}{*{20}c} 1 \\ i \\ \end{array} } \right].$$

where $E_{in}$ denotes the Jones vector for the incident light of intensity $I$ with $$E_{in} = \sqrt {\left( {I/2} \right)} \left[ {\begin{array}{*{20}c} 1 & i \\ \end{array} } \right]^{T}$$.

The measured intensity values for each polarization channel $\left( {I_{\psi } } \right)$ can be written as:

5 $$I_{\psi } \propto \left| {E_{out,\psi } } \right|^{2} = E_{out,\psi } E_{out,\psi }^{*} .$$

Using Eq. (1–5), one can easily obtain the expressions for the light intensity measured along the four polarization channels as follows:

6 $$\left( {\begin{array}{*{20}c} {\begin{array}{*{20}c} {I_{0^\circ } } \\ {I_{45^\circ } } \\ \end{array} } \\ {\begin{array}{*{20}c} {I_{90^\circ } } \\ {I_{135^\circ } } \\ \end{array} } \\ \end{array} } \right) = \frac{I}{2}\left( {\begin{array}{*{20}c} {\begin{array}{*{20}c} {J_{0^\circ , xx}^{2} + J_{0^\circ , xy}^{2} } \\ {J_{45^\circ , xx}^{2} + 2 J_{45^\circ , xy}^{2} + J_{45^\circ , yy}^{2} } \\ \end{array} } \\ {\begin{array}{*{20}c} {J_{90^\circ , xy}^{2} + J_{90^\circ , yy}^{2} } \\ {J_{135^\circ , xx}^{2} + 2 J_{135^\circ , xy}^{2} + J_{135^\circ , yy}^{2} } \\ \end{array} } \\ \end{array} } \right) = \frac{I}{2}\left( {\begin{array}{*{20}c} {\begin{array}{*{20}c} {1 - \sin \delta \sin 2\theta } \\ {1 + \sin \delta \cos 2\theta } \\ \end{array} } \\ {\begin{array}{*{20}c} {1 + \sin \delta \sin 2\theta } \\ {1 - \sin \delta \cos 2\theta } \\ \end{array} } \\ \end{array} } \right).$$

Then, we combine Eq. (6) to introduce two auxiliary quantities $Q_{1}$ and $Q_{2}$ defined as:

7 $$\left( {\begin{array}{*{20}c} {Q_{1} } \\ {Q_{2} } \\ \end{array} } \right) = \left( {\begin{array}{*{20}c} {\frac{{I_{90^\circ } - I_{0^\circ } }}{{I_{90^\circ } + I_{0^\circ } }}} \\ {\frac{{I_{135^\circ } - I_{45^\circ } }}{{I_{135^\circ } + I_{45^\circ } }}} \\ \end{array} } \right) = \left( {\begin{array}{*{20}c} {\sin \delta \sin 2\theta } \\ {\sin \delta \cos 2\theta } \\ \end{array} } \right).$$

Finally, the spatial distribution of retardance magnitude and the optic-axis orientation of the birefringent sample can be reconstructed as:

8 $$\delta \left( {x,y} \right) = \sin^{ - 1} \sqrt {Q_{1}^{2} + Q_{2}^{2} } ,$$

9 $$\theta \left( {x,y} \right) = \frac{1}{2}\tan^{ - 1} (\frac{{Q_{1} }}{{Q_{2} }}).$$

### Comparison of various demosaicing methods

**Figure 5:**
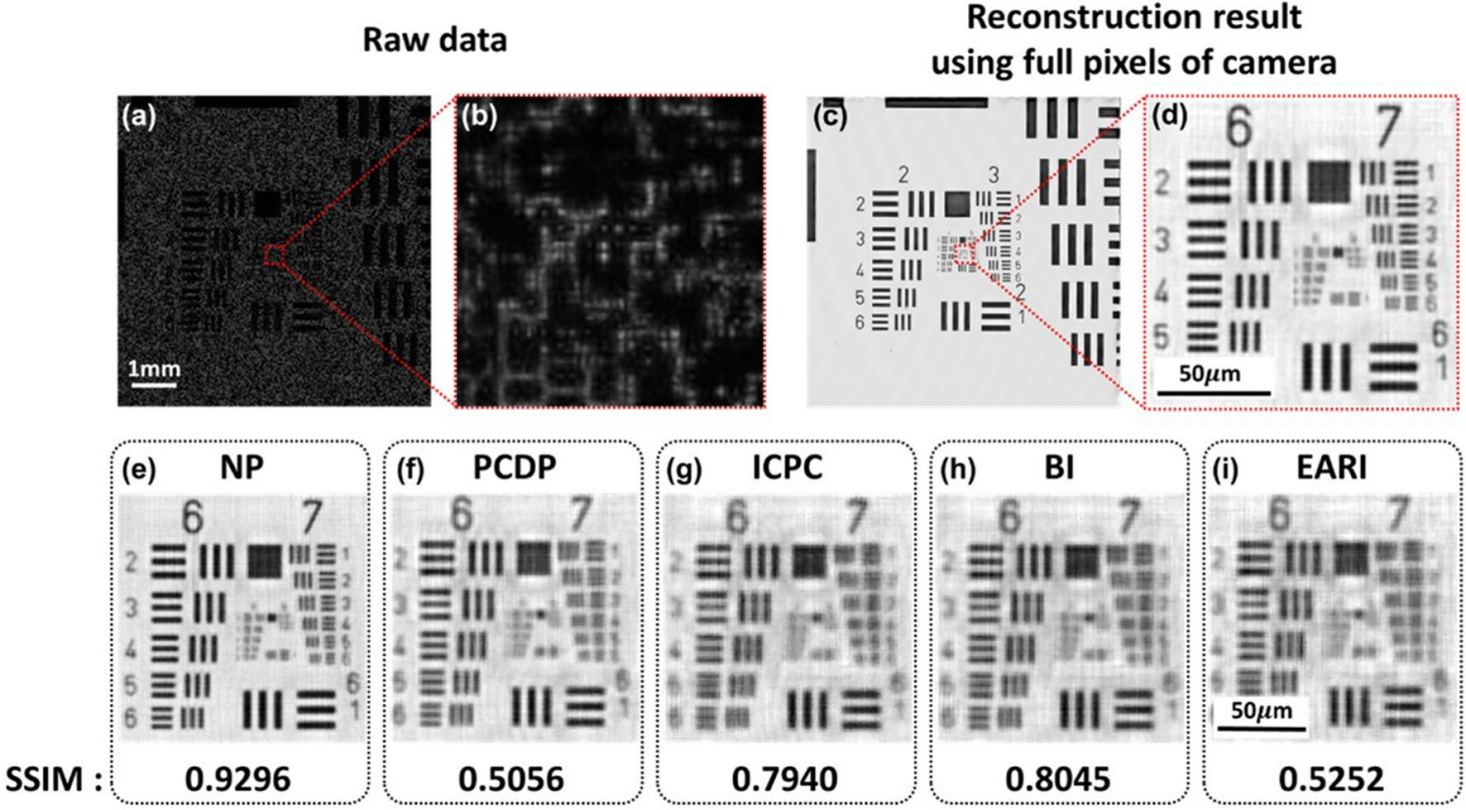
Compared image reconstruction performances of various pixel demosaicing methods. Compared image reconstruction performances of various pixel demosaicing methods. (**a**) Representative raw image of an USAF resolution target captured with an LED along the optical axis. (**b**) Enlarged image of the area marked with a red box in (**a**). (**c**) Reconstructed image of the target using the full pixels of the camera (i.e., without pixel demosaicing method). (**d**) Enlarged image of the area marked with a red box in (**c**). (**e**–**i**) Reconstruction results with various demosaicing methods. The acquired image was decomposed into the ones of each polarization channel and missing pixel information was estimated through (**e**) Newton’s polynomial (NP), (**f**) polarization channel difference prior (PCDP), (**g**) intensity correlation among polarization channels (ICPC), (**h**) bilinear interpolation (BI), and (**i**) edge-aware residual interpolation (EARI) algorithms, respectively. The structural similarity index measure (SSIM) values for each reconstruction result were evaluated against the result in (**c**) and are presented.

In order to find a suitable demosaicing method for PS-PtychoLM, we evaluated various pixel demosaicing methods including Newton’s polynomial (NP)^43^, polarization channel difference prior (PCDP)^68^, intensity correlation among polarization channels (ICPC)^69^, bilinear interpolation (BI), and edge-aware residual interpolation (EARI) algorithms^70^. We performed PS-PtychoLM imaging of an USAF resolution target and compared the reconstruction performances with the aforementioned demosaicing methods. A representative raw intensity image captured using the PS-PtychoLM is shown in Fig. 5a, along with an enlarged image of the area marked with a red box in Fig. 5a (Fig. 5b). As can be noted, the features in the object could not clearly be discerned because the object information was largely obscured by the mask-modulated object wave. Upon the acquisition of 81 images with angle-varied LED illuminations, we separated each image into the ones in four polarization channels and the missing pixel information in each channel was estimated using the interpolation methods. We also carried out image reconstruction using the full pixel information of the camera without demosaicing as the reference. Figure 5c,d present the image reconstruction results obtained using the full pixel information of the image sensor. It is evident that, after ptychographic retrieval, the features in the target can be clearly visualized. The reconstruction results using NP, PCDP, ICPC, BI, and EARI interpolation algorithms are presented in Fig. 5e–i, along with their structural similarity index measure (SSIM) values computed with reference to the result in Fig. 5c. It can be noted that the NP interpolation method provided superior image reconstruction compared to other methods. This superior performance of NP compared with others may be accounted for by that the NP operates on the polynomial interpolation error estimation, thus being much effective in preserving both low- and high-spatial-frequency information^43^.

### Supplementary Information

Supplementary Information.

## Data Availability

All the data are available upon reasonable request to the corresponding author (cjoo@yonsei.ac.kr).
