# Supplementary material for: Ptychographic lens-less birefringence microscopy using a mask-modulated polarization image sensor
Source: Sci Rep. 2023 Nov 7;13:19263. doi: 10.1038/s41598-023-46496-z (PMC10630341; doi:10.1038/s41598-023-46496-z)
Supplement: Supplementary file 1 — Supplementary Information. [file 41598_2023_46496_MOESM1_ESM.pdf]

Supplementary Information

Ptychographic lens-less birefringence microscopy using a mask-modulated polarization image sensor

Jeongsoo Kim[1], Seungri Song [1], Hongseong Kim[1], Bora Kim[2], Mirae Park[3], Seung Jae Oh[3,4], Daesuk Kim[5], Barry Cense[1,6], Yong-min Huh[3,4,7], Joo Yong Lee[2], and Chulmin Joo[1]*

[1] Department of Mechanical Engineering, Yonsei University, 03722, Seoul, Republic of Korea

[2] Department of Ophthalmology, Asan Medical Center, University of Ulsan College of Medicine, 05505, Seoul, Republic of Korea

[3] Department of Radiology, College of Medicine, Yonsei University, 03722, Seoul, Republic of Korea

[4] YUHS-KRIBB Medical Convergence Research Institute, 03722, Seoul, Republic of Korea

[5] Department of Mechanical System Engineering, Jeonbuk National University, 54896, Jeonju, Republic of Korea

[6] Department of Electrical, Electronic & Computer Engineering, The University of Western Australia, Perth, WA 6009, Australia

[7] Department of Biochemistry & Molecular Biology, College of Medicine, Yonsei University, 03722, Seoul, Republic of Korea

* Corresponding author: Chulmin Joo (cjoo@yonsei.ac.kr).

Section 1. PS-PtychoLM forward model

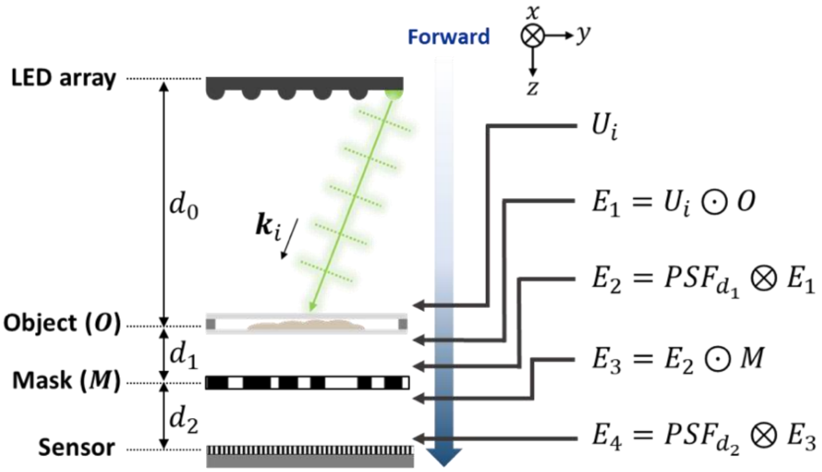

Figure S1. PS-PtychoLM image formation. $\boldsymbol{U_i}$, $\boldsymbol{O}$, and $\boldsymbol{M}$ represent the incident light field from the $\boldsymbol{i^{th}}$ LED element, complex information of the object, and optical mask, respectively. $\boldsymbol{d_0}, \boldsymbol{d_1}$, and $\boldsymbol{d_2}$ denote the distances between the LED array and object, the object to the mask, and the mask to sensor plane, respectively. The spatial coordinate $\boldsymbol{r}$ is omitted for simplicity.

The forward imaging model of PS-PtychoLM was used to predict the manner in which the optical wave passing through the object and mask was recorded at the image sensor (Figure S1). Consider an object illuminated by the ith element in the LED array. In the implementation, the distance between the LED elements and the object was large (~400mm), and thus the illumination light wave from the ith LED element (U_i) can be assumed to be a plane wave in the object plane, which is expressed as:

$$U_i(\boldsymbol{r}) = \exp[j\boldsymbol{k_i} \cdot \boldsymbol{r}], \tag{1}$$

where $\boldsymbol{r} = (x, y)$ denotes the spatial coordinate, j is the imaginary unit, $\boldsymbol{k_i} = (k_{x,i}, k_{y,i})$ is the incident wave vector, and $\cdot$ is the inner product of vectors. Using the PS-PtychoLM configuration (Figure S1), the incident wave can be re-written as $k_{x,i} = -ksin\,[tan^{-1}(x_{LED,i}/d_0)]$, $k_{y,i} = -ksin\,[tan^{-1}(y_{LED,i}/d_0)]$, where k is the wave number of light and $(x_{LED,i}, y_{LED,i})$ is the lateral location of the ith LED element. The illumination field then interacts with the object and the exit light field in the object plane can be written as $E_1(\boldsymbol{r}) = U_i(\boldsymbol{r}) \odot O(\boldsymbol{r})$, where $O(\boldsymbol{r})$ is the object complex information and $\odot$ stands for the element-wise multiplication. The exit wave E_1 then propagates to the mask plane and the wave incident on the mask is given by $E_2(\boldsymbol{r}) = PSF_{d_1} \otimes E_1(\boldsymbol{r})$, where PSF_d represents the point spread function (PSF) for free-space propagation over the propagation distance d, and $\otimes$ denotes a convolution operation. In the implementation, free-space propagation was operated in the Fourier space using the angular spectrum method[1-3]. The light field E_2 is modulated by the optical mask, resulting in $E_3(\boldsymbol{r}) = E_2(\boldsymbol{r}) \odot M(\boldsymbol{r})$, where $M(\boldsymbol{r})$ is the mask transmission function. The modulated light field E_3 further propagates to the sensor plane to result in $E_4(\boldsymbol{r}) = PSF_{d_2} \otimes E_3(\boldsymbol{r})$. The intensity image captured by the image sensor with the ith LED illumination (I_i) can then be written as:

$$I_i(\boldsymbol{r}) = |E_4(\boldsymbol{r})|^2 = \left|PSF_{d_2} \otimes [PSF_{d_1} \otimes [U_i(\boldsymbol{r}) \odot O(\boldsymbol{r})] \odot M(\boldsymbol{r})]\right|^2. \tag{2}$$

Section 2. PS-PtychoLM image reconstruction procedure

By sequentially turning on each LED, a set of intensity images I_i ($i = 1, 2, \dots, L$) is acquired and used for PS-PtychoLM image reconstruction. In our demonstration, we recorded 81 intensity images with a size of $M \times M$ by turning on the 81 LEDs centered at the optical axis. In order to reconstruct complex information of object and binary mask, we used the regularized ptychographic iterative engine (rPIE)[4] and the reciprocal-space up-sampling method. A detailed reconstruction procedure is presented as follows.

Step 1) We first initialize the object and mask as constant all-one matrix with a size of $(N \times M) \times (N \times M)$, where N represents the up-sampling ratio. Under the ith LED illumination, the field at the sensor plane (E_4) can be estimated using the PS-PtychoLM forward model. Since the recorded intensity image (I_i) has a size $M \times M$, the estimated E_4 is down-sampled with a factor of N, then the information is updated with the acquired intensity image and then up-sampled again by N-times. This process is expressed as follows:

$$E_4' = E_4(\boldsymbol{r}) \odot \uparrow_N \left\{ \sqrt{\frac{I_i}{\downarrow_N |E_4(\boldsymbol{r})|^2 \otimes ones(N \times N)}} \right\}, \tag{3}$$

where $\uparrow_N$ () and $\downarrow_N$ () denote the *N*-times nearest-neighbor up-sampling and down-sampling operations, respectively. $ones(N \times N)$ represents an $N \times N$ all-one matrix. The convolution procedure in the denominator of Eq. (3) using $ones(N \times N)$ enforces the intensity sum of each $N \times N$ segment of the result to be equal to the corresponding pixel of the acquired image[5]. Note that, in our implementation, the $N \times N$ all-one matrix was zero-padded to the size $(N \times M) \times (N \times M)$ and then the convolution operation was performed in the Fourier domain. The value of N was set to 4.

Step 2) The updated light field E_4' is then back-propagated to the mask plane as:

$$E_3'(\boldsymbol{r}) = conj\left(PSF_{d_2}\right) \otimes E_4'(\boldsymbol{r}), \tag{4}$$

where $conj()$ represents complex conjugation.

Step 3) Complex information of the mask and light field above the mask is updated using the rPIE algorithm as:

$$M'(\boldsymbol{r}) = M(\boldsymbol{r}) + \beta \cdot \frac{conj[E_2(\boldsymbol{r})]}{(1-\alpha)[|E_2(\boldsymbol{r})|^2] + \alpha \cdot \max_{\boldsymbol{r}}[|E_2(\boldsymbol{r})|^2]} \odot [E_3'(\boldsymbol{r}) - E_3(\boldsymbol{r})], \tag{5}$$

$$E_2'(\boldsymbol{r}) = E_2(\boldsymbol{r}) + \beta \cdot \frac{conj[M'(\boldsymbol{r})]}{(1-\alpha)[|M'(\boldsymbol{r})|^2] + \alpha \cdot \max_{\boldsymbol{r}}[|M'(\boldsymbol{r})|^2]} \odot [E_3'(\boldsymbol{r}) - E_3(\boldsymbol{r})], \tag{6}$$

where α and β are the weighting parameters of the rPIE algorithm. We set $\alpha = 0.8$ and $\beta = 0.5$ in the implementation.

Step 4) The updated light field E_2' is then back-propagated to the object plane:

$$E_1'(\boldsymbol{r}) = conj\left(PSF_{d_1}\right) \otimes E_2'(\boldsymbol{r}). \tag{7}$$

Step 5) Lastly, the complex information of object is updated with the light field E_1' as:

$$O'(\boldsymbol{r}) = O(\boldsymbol{r}) + \beta \cdot \frac{conj[U_i(\boldsymbol{r})]}{(1-\alpha)[|U_i(\boldsymbol{r})|^2] + \alpha \cdot \max_{\boldsymbol{r}}[|U_i(\boldsymbol{r})|^2]} \odot [E_1'(\boldsymbol{r}) - E_1(\boldsymbol{r})]. \tag{8}$$

The object O and mask M are replaced with the updated object O' and mask M' before starting the update procedures (steps 1-5) using the LEDs in different positions. One iteration is complete if the update procedure is performed for all the LEDs. When the object and mask converge, the reconstruction is finished, and in our experiment, it generally took approximately 10 iterations to complete.

In our PS-PtychoLM implementation, a binary mask with a pre-defined pattern was employed for robust image reconstruction[6]. Even though the mask pattern is pre-defined and fabricated with high fidelity, it is extremely challenging to align the mask in a designated space in the optical setup. In practice, the actual mask pattern that contributes to the image formation may have a lateral shift and rotation, and it may be a small portion of the designed mask pattern. This uncertainty on the actual mask pattern may result in a complete failure of image recovery. We obtained the mask pattern that contributed to the image formation with the three steps outlined below (Figure S2):

Step 1) We first perform a blind recovery using a guessed mask pattern. The objective of this blind recovery is to obtain a rough estimate of the mask pattern. In this step, the complex object information and the mask pattern are initialized by all-one matrix, respectively. Then, one iteration of image recovery is conducted, and then rough estimates of the mask pattern and the object are obtained.

Step 2) We then conduct cross-correlation of the estimated mask pattern obtained from Step 1 and the designed mask pattern to determine the amount of lateral shift and rotation of the actual mask with respect to the designed mask pattern. The resultant information was used to further refine the actual mask pattern that contributes to the image formation.

Step 3) The obtained actual mask pattern is then used as the initial guess for the subsequent iteration of image recovery.

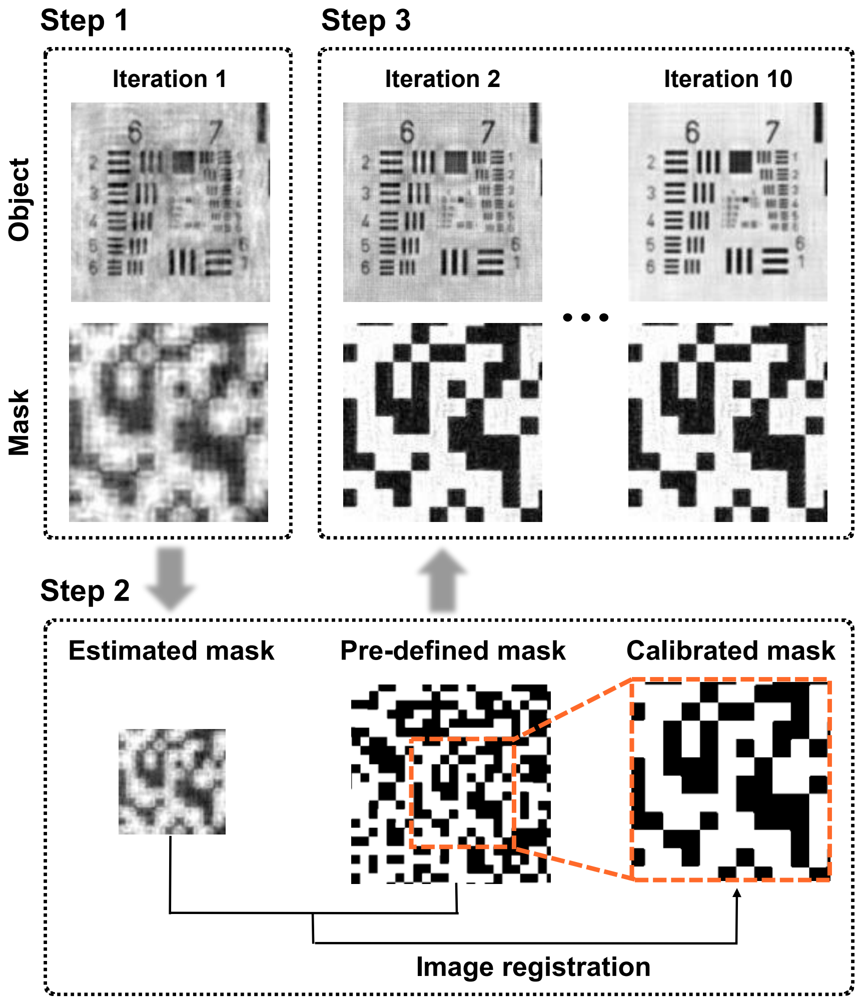

Figure S2. Procedures to obtain the actual mask pattern in the PS-PtychoLM image reconstruction.

Section 3. Evaluated PS-PtychoLM imaging performance

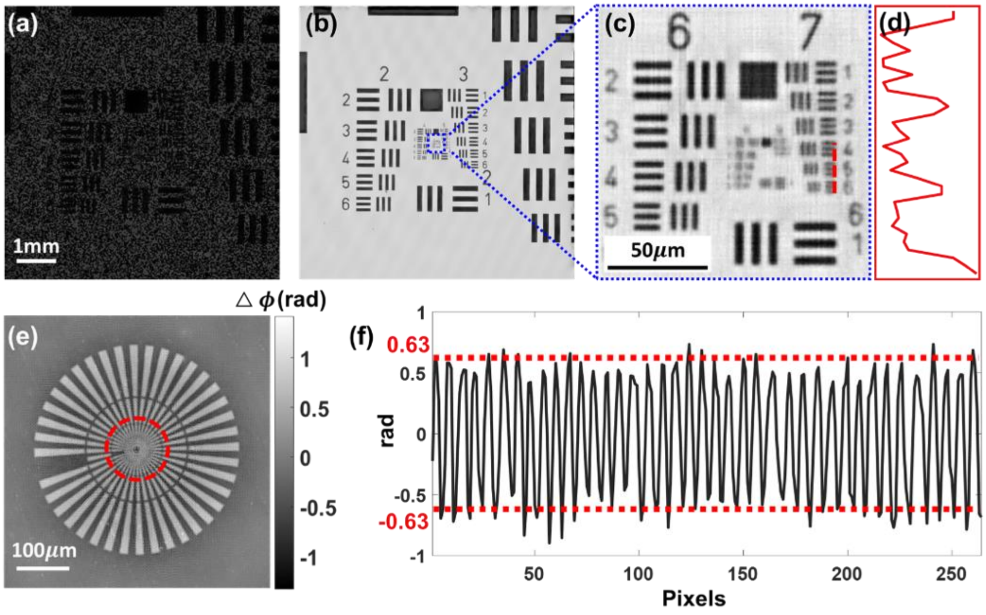

Figure S3. PS-PtychoLM imaging performance evaluation. (a-d) USAF 1951 resolution target imaging results. (a) Representative PS-PtychoLM raw image of the target. (b) Reconstructed USAF 1951 resolution target image. (c) Enlarged image of the area marked with a blue box in (b). (d) Line profile along dashed red line in (c). (e,f) Quantitative phase target imaging result. (e) Reconstructed phase image. (f) Phase profile along the dashed red circle in (e).

In order to evaluate the imaging performance of PS-PtychoLM, we first imaged a USAF 1951 resolution target (Edmund optics, #64-862) (Figure S3a-d). An exemplary raw image captured by the PS-PtychoLM is shown in Figure S3a. After the ptychographic reconstruction with 81 image acquisitions, an amplitude image of the resolution target over the entire FoV of 7.07mm x 8.45mm could be reconstructed (Figure S3b). One can see that features in the resolution target can be clearly visualized. Figure S3c is an enlarged image of the area marked with a blue box in Figure S3b. The element 5 in group 7 (line width = 2.46 µm) could be distinguished. The line profile along the dashed red line in Figure S3c is shown in Figure S3d. Our PS-PtyhcoLM prototype achieved a half-pitch resolution of 2.46 µm for a FoV of 7.07 mm x 8.45 mm, which corresponds to an SBP of 9.9 megapixels.

The phase reconstruction performance was then evaluated using a quantitative phase target (Benchmark, Quantitative phase target). Figure S3e depicts the reconstruction result of the phase target using the PS-PtychoLM platform. The phase distribution along the dashed red circle in Figure S3e is presented in Figure S3f. The target features in the reconstructed area shown in Figure S3e are characterized by a height of 200 nm and a refractive index of 1.52. The theoretical phase value, computed from this information and the LED center wavelength (514.5 nm), is indicated by a red dashed line in figure S3f. The reconstructed phase value agrees with the actual value with an error of 6.15%.

Section 4. Birefringence reconstruction results with random diffuser and binary mask

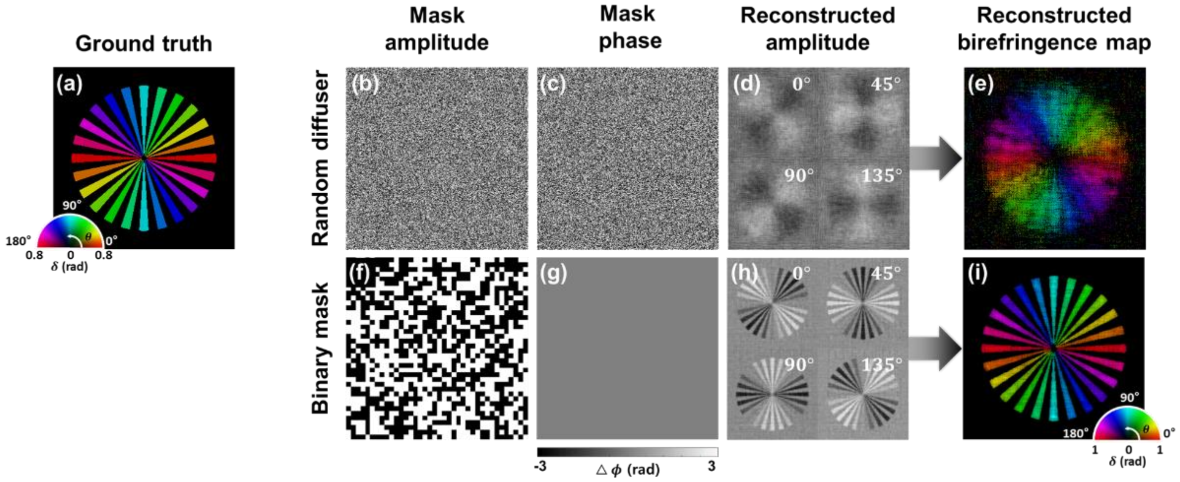

Figure S4. Comparison of the PS-PtychoLM birefringence reconstruction performance with an unknown random diffuser and pre-defined binary mask. (a) Ground truth birefringence information of a simulation target. (b,f) Amplitude and (c,g) phase information of the random diffuser and binary mask, respectively. (d,h) Reconstructed amplitude information of each polarization channel and (e,i) obtained birefringence map using the random diffuser and binary mask, respectively.

We employed a pre-defined binary structure as the modulation mask in our PS-PtychoLM implementation. This mask served as a constraint in the iterative phase retrieval process, which helps, along with the captured images, convergence to the solution. We separately evaluated a random mask with unknown micro-/nano- features to examine if it can be used in our PS-PtychoLM imaging. The random mask has been employed in various forms of lens-less ptychographic microscopes to achieve higher spatial resolution[7,8]. Our results are presented in Figure S4. We considered a birefringence object depicted in Figure S4a as the target and performed numerical simulations with an unknown random diffuser and binary mask. Figure S4b,c present the amplitude and phase distributions of the random diffuser used in the simulation. The feature size of the diffuser was set to be 3.45 μm, and its amplitude and phase values were uniformly distributed in the interval [0, 1] and [$-\pi$, π], respectively. The reconstructed amplitude images of each polarization channel and birefringence information are shown in Figure S4d,e. The amplitude information of the target could not be reconstructed accurately, and consequently, the reconstructed birefringence map also significantly deviated from the ground truth. This can be explained in part by that the random diffuser significantly scrambles the propagation wavefront of the object exit wave, and thus polarization states in the missing pixels could not be correctly interpolated by Newton's polynomial demosaicing method.

In contrast, the use of a pre-determined binary mask with a mean feature size of 27.6 μm enabled an accurate reconstruction of the object birefringence information. Figure S4f,g are the amplitude and phase of the binary mask used in the simulation, respectively. The amplitude of the mask consisted only of 0 and 1, and the phase was constant across the plane (phase = 0 in this simulation). The reconstructed amplitude images of each polarization channel and birefringence map clearly demonstrate that the binary mask enabled accurate estimation of the polarization information throughout all polarization channels, thus resulting in accurate birefringence map.

References

1. Goodman, J. W. *Introduction to Fourier optics*. (Roberts and Company publishers, 2005).

2. Matsushima, K. & Shimobaba, T. Band-limited angular spectrum method for numerical simulation of free-space propagation in far and near fields. *Optics express* **17**, 19662-19673 (2009).

3. Goldstein, D. H. *Polarized light*. (CRC press, 2017).

4. Maiden, A., Johnson, D. & Li, P. Further improvements to the ptychographical iterative engine. *Optica* **4**, 736-745 (2017).

5. Batey, D. *et al.* Reciprocal-space up-sampling from real-space oversampling in x-ray ptychography. *Physical Review A* **89**, 043812 (2014).

6. Lu, C. *et al.* Mask-modulated lensless imaging via translated structured illumination. *Optics Express* **29**, 12491-12501 (2021).

7. Jiang, S. *et al.* Wide-field, high-resolution lensless on-chip microscopy via near-field blind ptychographic modulation. *Lab on a Chip* **20**, 1058-1065 (2020).

8. Jiang, S. *et al.* Resolution-enhanced parallel coded ptychography for high-throughput optical imaging. *ACS Photonics* **8**, 3261-3271 (2021).
